# Glucose management for exercise using continuous glucose monitoring: should sex and prandial state be additional considerations? Reply to Yardley JE and Sigal RJ [letter]

**DOI:** 10.1007/s00125-020-05374-3

**Published:** 2021-02-04

**Authors:** Othmar Moser, Michael C. Riddell, Max L. Eckstein, Peter Adolfsson, Rémi Rabasa-Lhoret, Louisa van den Boom, Pieter Gillard, Kirsten Nørgaard, Nick S. Oliver, Dessi P. Zaharieva, Tadej Battelino, Carine de Beaufort, Richard M. Bergenstal, Bruce Buckingham, Eda Cengiz, Asma Deeb, Tim Heise, Simon Heller, Aaron J. Kowalski, Lalantha Leelarathna, Chantal Mathieu, Christoph Stettler, Martin Tauschmann, Hood Thabit, Emma G. Wilmot, Harald Sourij, Carmel E. Smart, Peter G. Jacobs, Richard M. Bracken, Julia K. Mader

**Affiliations:** 1 Division of Exercise Physiology and Metabolism, Department of Sport Science, University of Bayreuth, Bayreuth, Germany; 2 Division of Endocrinology and Diabetology, Department of Internal Medicine, Medical University of Graz, Graz, Austria; 3 School of Kinesiology and Health Science, York University, Toronto, ON Canada; 4 Department of Pediatrics, The Hospital of Halland, Kungsbacka, Sweden; 5 Sahlgrenska Academy at University of Gothenburg, Institution of Clinical Sciences, Gothenburg, Sweden; 6 Institut de Recherches Cliniques de Montréal, Montréal, QC Canada; 7 Endocrinology Division Centre Hospitalier Universitaire de Montréal, Montréal, QC Canada; 8 Nutrition Department, Faculty of Medicine, Université de Montréal, Montréal, QC Canada; 9 Montreal Diabetes Research Centre, Montréal, QC Canada; 10 Division of Pediatric Diabetology, DRK Children’s Hospital, Siegen, Germany; 11 Department of Endocrinology, University Hospitals Leuven, KU Leuven, Leuven, Belgium; 12 Steno Diabetes Center Copenhagen, University of Copenhagen, Copenhagen, Denmark; 13 Department of Metabolism, Digestion and Reproduction, Faculty of Medicine, Imperial College, London, London, UK; 14 Department of Pediatric Endocrinology and Diabetes, Stanford University School of Medicine, Stanford, CA USA; 15 Department of Paediatric Endocrinology, Diabetes and Metabolic Diseases, UMC - University Children’s Hospital, University Medical Centre Ljubljana, Ljubljana, Slovenia; 16 Faculty of Medicine, University of Ljubljana, Ljubljana, Slovenia; 17 Department of Pediatric Diabetes and Endocrinology, Centre Hospitalier Luxembourg, Luxembourg, Luxembourg; 18 Department of Pediatrics, Free University Brussels (VUB), Brussels, Belgium; 19 International Diabetes Center, Minneapolis, MN USA; 20 Department of Pediatrics, Yale School of Medicine, New Haven, CT USA; 21 Bahçeşehir Üniversitesi, Istanbul, Turkey; 22 Paediatric Endocrinology Division, Shaikh Shakhbout Medical City, Abu Dhabi, United Arab Emirates; 23 Profil, Neuss, Germany; 24 Department of Oncology & Metabolism, The Medical School, University of Sheffield, Sheffield, UK; 25 Sheffield Teaching Hospitals NHS Foundation Trust, Sheffield, UK; 26 JDRF, New York, NY USA; 27 Manchester Diabetes Centre, Manchester University NHS Foundation Trust, Manchester Academic Health Science Centre, Manchester, UK; 28 Department of Diabetes, Endocrinology and Gastroenterology, Faculty of Biology, Medicine and Health, University of Manchester, Manchester, UK; 29 Department of Diabetes, Endocrinology, Nutritional Medicine and Metabolism, Inselspital, Bern University Hospital and University of Bern, Bern, Switzerland; 30 Department of Pediatrics and Adolescent Medicine, Medical University of Vienna, Vienna, Austria; 31 Diabetes Department, Royal Derby Hospital, University Hospitals of Derby and Burton NHSFT, Derby, UK; 32 Faculty of Medicine & Health Sciences, University of Nottingham, Nottingham, UK; 33 School of Health Sciences, University of Newcastle, Callaghan, NSW Australia; 34 Department of Paediatric Diabetes and Endocrinology, John Hunter Children’s Hospital, Newcastle, NSW Australia; 35 Department of Biomedical Engineering, Oregon Health & Science University, Portland, OR USA; 36 Applied Sport, Technology, Exercise and Medicine Research Centre (A-STEM), College of Engineering, Swansea University, Swansea, UK

**Keywords:** Carbohydrates, CGM, Exercise, Physical activity, Type 1 diabetes

*To the Editor:* We thank Dr Yardley and Dr Sigal for their comments on the position statement pertaining to the use of continuous glucose monitoring (CGM) systems around exercise in type 1 diabetes [1, 2]. The letter by Yardley and Sigal was centred on four points: (1) the potential difference in risk of hypoglycaemia in active vs inactive people; (2) possible sex differences in exercise-related hypoglycaemia risk; (3) the role of bolus insulin administration to prevent and/or correct exercise-related hyperglycaemia that is sometimes associated with high-intensity interval exercise (HIIE) and/or resistance exercise; and (4) the role of the prandial state on glucose responses during exercise.

As Yardley and Sigal state [1], the role of fitness level and/or exercise experience on the risk for hypoglycaemia during or after exercise is unclear. To clarify, our position statement did not mention that ‘inactive’ people with type 1 diabetes have a higher risk of hypoglycaemia per se; however, it was discussed that ‘individuals who do not routinely exercise may face an increased risk of hypoglycaemia’, ‘partially’ in line with the study of Bohn et al [2, 3]. Yardley and Sigal reference the article by Al Khalifah et al [4] as evidence that people who are more aerobically fit may have a higher likelihood of becoming hypoglycaemic during exercise compared with those less fit. A closer look at the data from this paper, however, indicates that there was a much larger number of participants in the ‘good fitness’ group who performed treadmill exercise (*n* = 19) compared with exercise on the stationary bike (*n* = 4), whereas, in the ‘poor fitness’ group, a larger number of participants performed exercise on the stationary bike (*n* = 11) compared with the treadmill (*n* = 9). A larger percentage of hypoglycaemia occurred across both groups with the treadmill exercise compared with exercise on the stationary bike, perhaps partly because the treadmill exercise was twice as long as that on the stationary bike. For this reason, a number of confounders prevent a conclusion on the impact of fitness level on likelihood of hypoglycaemia based on these results. Based on the large cross-sectional study by Bohn et al [3] and in agreement with the present writing groups’ clinical experience, we would still recommend higher glucose levels during exercise for those that are not routinely exercising to ensure safe exercise and a low risk of hypoglycaemia. Importantly, if a person exercises routinely and/or has a low risk of hypoglycaemia over a period of 3 months, then the glycaemic thresholds for carbohydrate consumption might be adjusted, as emphasised in our statement [2].

Thank you for discussing several studies showing how hypoglycaemia risk during exercise can be different for male individuals vs female individuals. In Fig. 1 of the manuscript by Bohn et al, severe hypoglycaemia was demonstrated to occur more often in physically active female participants compared with male participants; however, the difference was small (two events per 100 person-years) [3]. Brockman et al [5] showed that it was male individuals, not females, who experienced the sharper decrease in glucose during resistance exercise and also higher rates of hypoglycaemia. Given the conflicting results, small differences between the groups and the limited studies in this area, we provided an initial focus on the overall population of people with type 1 diabetes, separating them into adults, adolescents and children, with subgroups based on risk of hypoglycaemia [2]. While we anticipate that future studies will provide a more complete picture on the role of sex in modulating exercise-induced changes in blood glucose levels, our position statement [2] is a first step towards evidence-based general recommendations about how to use CGM systems around exercise.

The authors mention in their letter that we recommend a bolus insulin correction in advance of HIIE and resistance exercise. While dosing insulin in advance of HIIE and resistance exercise may be dangerous in some situations, for the points raised in our position statement [2], we recommended a 50% reduced correction factor of the regular insulin dose at glucose levels >10 mmol/l, and not an insulin correction dose before HIIE and resistance exercise in general (see Table 1 in the position statement [2]). We also note that pre-exercise extreme hyperglycaemia (sensor glucose levels >15 mmol/l) can be treated with an insulin bolus (50% of a typical correction dose) [2]. We maintain this recommendation, given the strong emphasis for the use of CGM in this position statement to allow for the safe monitoring of glucose levels post insulin correction [2]. We feel that it is unwarranted to recommend that people with type 1 diabetes ignore their pre-exercise hyperglycaemia prior to exercise or competition [6].

We agree with Yardley and Sigal that a determining factor of whether glucose increases during resistance exercise or HIIE may be dependent on the prandial state [1]. We agree that we may expect to see elevated glucose levels during resistance exercise and HIIE when done in the fasted state and addressed this point in the position statement, where we stated that ‘if sensor glucose is expected to increase, as often seen in people performing fasted high-intensity interval training, resistance training and, also, in training above the anaerobic threshold, then an insulin correction can be administered at the onset of, as well as during exercise (50% of typical correction factor)’ [2].

The four points discussed here highlight the multiple factors involved in intra- and interindividual glucose response to physical activity and exercise in individuals living with type 1 diabetes and, accordingly, the difficulties encountered by them in anticipating and managing glucose in these situations. Thus, we would like to thank the authors for their support on this position statement and appreciate the additional viewpoints that will be considered in future updates of a rapidly advancing area.

## Abbreviations

CGM: Continuous glucose monitoring
HIIE: High-intensity interval exercise
